# Epidemiologic Determinants of Dynamics in Heart Failure Prevalence and Mortality in Older U.S. Adults

**DOI:** 10.1093/geroni/igab046.625

**Published:** 2021-12-17

**Authors:** Bin Yu, Igor Akushevich, Arseniy Yashkin, Julia Kravchenko

**Affiliations:** 1 Duke University, Duke University/Durham, North Carolina, United States; 2 Duke University, Durham, North Carolina, United States; 3 Duke University, Morrisville, North Carolina, United States

## Abstract

Recent declines in heart failure (HF) prevalence and increases in mortality among older adults in the US suggest the need for research to investigate the relative contribution of the epidemiological determinants of these two processes to their historical and current trends. Study data were derived from a 5% sample of Medicare beneficiaries, 1991-2017. Partitioning analysis was used to decompose age-adjusted prevalence and incidence-based mortality (IBM) into their constituent components. HF prevalence trend decomposition demonstrated three phases: (a) Decelerated Increasing Prevalence (1994-2006) mainly driven by decreasing incidence, overpowering increasing survival, (b) Accelerated Declining Prevalence (2007-2014) and (c) Decelerated Declining Prevalence (2015-2017), mainly driven by declining incidence, overpowering declining survival. For HF IBM four phases were identified: (a) Decelerated Increasing Mortality (1994-2001) with declining incidence and increasing survival driving deceleration, (b) Accelerated Declining Mortality (2002-2012), (c) Decelerated Declining Mortality (2013-2016), mainly driven by declining incidence, overpowering declining survival, and (d) Accelerated Increasing Mortality (2017) mainly driven by declining survival, overpowering declining incidence. Study findings suggest that the recent decade-long decline in HF prevalence and 15-year decline in HF mortality mainly reflected decreasing incidence, while the most recent increase in mortality was due to declining survival, which may be associated with the Hospital Readmission Reduction Program. If current trends of incidence and survival persist, HF prevalence and mortality are forecasted to grow, suggesting that actions to reduce HF risk factors and improve treatment and management of HF after diagnosis are warranted.

